# Treatment of Exposed Pulps

**Published:** 1876-07

**Authors:** C. C. Chittenden

**Affiliations:** Madison.


### ARTICLE III

### *Treatment of Exposed Pulps.*

BY C. C. CHITTENDEN, OF MADISON.

Read before Wisconsin State Dental Society.

In undertaking to fulfil your committee's request to prepare and present a paper on the subject in question, I am actuated, not by the hope of imparting anything new or startling, but rather by the desire to provoke discussions, and gather therefrom for my own instruction and use in practice.

Therefore, with no attempt to present a paper, burdened down with its own weight of originality, I have put in form of words a few thoughts which are the results of my own daily experience in combating with the diseases in question.

The general practitioner in dentistry is daily called upon to extract teeth for the relief of pain and suffering, which if he is properly educated and posted in his calling, he is quite certain need not be lost, and which, if he is honest to himself, he will refuse to extract.

Fully one half of these cases are of exposed pulps, or at least of teeth whose pulp chambers are so nearly approached by caries of the tooth-bone as to involve the health, if not the life of the dental pulp. Premising that treatment of the dental pulp includes its destruction and extirpation, as well as its salvation, we will proceed to consider what the dentist ought to do when such cases are presented.

His plain duty is first to carefully investigate the extent of caries, as to the chances of permanently building and restoring, (by filling,) the lost portions of the tooth, in the event of relieving the more pressing trouble of pain. This step in diagnosis is of vital importance, and should not be carelessly or hastily decided, especially in the negative.

The simple fact that every tooth untouched by disease is possessed of a living pulp, is the best evidence that should be retained alive, if within the possibilities. While advocating radical measures in general operative practice, we should practice conservatism to a radical degree, where the dental pulp is involved, for the reason that after exhausting every resource to save, it may be deadened and removed as easily, and with as good chance of saving the tooth as if no effort to save the pulp had been made. Decide then—can the pulp be saved alive?

The chief points to be considered are, First—condition of the pulp and the extent to which it is involved. Second

—age of patient. Third—treatment. Fourth—condition of general health.

The pulp may be, to all extents and purposes regarding the involvement of its health and life, exposed, while yet there remains enough of original dentinal structure covering it to admit of the careful removal of all decalcified matter in the cavity, without actually coming in contact with it. If this is the case, or if there is actual exposure of recent date, with no great amount of irritation of its tissue, we may reasonably hope for success; but if, on the other hand, exposure has existed for some time, irritation is extensive, and pain therefrom long continued, the other points enumerated above must be carefully considered.

The age of the patient will have considerable weight as to chances of success. In the undeveloped child or youth, and the adult past the meridian of life, the greater involvement is very apt to result in loss of life of the pulp. While in the child and youth, in case of recent exposure, there is reasonable hope of Nature's protecting by an extra deposit of bone material if assisted, and while in advanced life Nature may have power enough left to come to the rescue, yet in the fully-rounded and full powered adult may we hope most.

The temperament of patient has of course its bearing upon the results hoped to be attained.

The sanguine, if coupled with an inflammatory diathesis, is most apt to prove a stumbling block to success, for when in this temperament there has been any considerable irritation of pulp, inflammation and congestion are apt to ensue in spite of the most careful treatment. In the lymphatic and encephalic or melancholic temperaments, success may often be obtained, but from my observation, the pulp is here more liable to *seem* to live, while in reality, it sooner or later quietly "dies and makes no sign," and on after examination will be found to have, to more or less extent, guarded itself, but finally shrivelled and left the chambers and canals empty and clean. These cases generally give no aftertrouble, and the last named result is only brought to light by accident.

The nervous in its combinations with the sanguine and the billious, *i. e.* the nervo-sanguine and nervo-billious temperaments are those which promise to give best results. I can trace more cases of real success to them than to any others. This matter of temperaments is of more importance than would at first appear, and has, I firmly believe, great weight in the chances of saving exposed dental pulps.

Physical health certainly, is most desirable in treatment of this as well as any other local difficulty, and its impairment will certainly militate against ultimate success.

A third condition of exposed pulp, and one not hitherto spoken of, is that when the tissue has become so far involved that a portion of the pulp has lost its vitality, or its nature and functions have lost their normal condition and action. Whether this be indicated by abnormal secretion, or by expansion beyond the limits of its chamber into the decayed cavity, or yet by decided congestion, involving the peridental membrane, it is generally best to proceed at once to destroy and remove the pulp.

But to return.

Having decided that the pulp can be saved, the question arises, how best to treat it to that end?

If the exposure is slight and irritation limited, a thorough bath of pure creosote is generally all sufficient before applying the capping, yet even here it is safer to move slowly than to cap in haste. In the more complicated cases, careful treatment is of the greatest importance. I know it is generally prescribed to bathe with creosote, cap, and wait results. But my experience is that to follow this line of treatment literally, is too often a sure way of being "in at the death" of the pulp. Rather let it die in spite of than because of your treatment. In every case, relief from pain and uneasiness should always be secured before capping.

To cap a pulp while diseased or irritated, means to destroy its life. The point to be attained is described by Dr.

Johnson, who said the object of medicine was to entertain the disease, while Nature performed the cure. To entertain the disease in this instance means to remove the cause of irritation. The uncovered pulp is of course subjected to more or less contact with the fluids of the mouth, as well as to particles of food lodged in the decayed tooth, which are always, to a greater or less degree, in a state of decomposition. Therefore the cavity should be carefully cleansed of foreign matter and diseased tooth bone, so far as it can be accomplished, without producing pain by mechanical means.

Treatment for disinfection and relief of irritation should be applied, and the cavity sealed for a few days, then opened, and if necessary, (which is rarely) the same process repeated until the pulp is restored, by being then placed beyond the influence of anything likely to provoke inflammation and consequent congestion. The remedies or "entertainers" are various and numerous; one proving most successful in the hands of one operator, and another in those of another, that most successful in my hands being creosote and tannin.

Nature having been thus assisted to perform a cure, it but remains to guard the exposed point from liability to repetition of this trouble by placing a safeguard or capping over it, and then properly filling the cavity.

Of the different materials yet used for capping, I will speak only of the two which seem to possess greatest merit, viz.: Gutta-percha and the Zinc oxy-chloride.

The former, when it can be used without danger of being forced into the chamber by the introduction of the gold filling, is preferable to the zinc, on account of the absence of the irritating property possessed by the latter. But when the zinc can be introduced without producing pain, *it* is certainly preferable to anything in use, by reason of its density and antiseptic properties.

After capping, when circumstances will permit, some weeks should be allowed to elapse before subjecting the tooth to the severe exercise of the mallet in building a permanent filling.

Should we, after doing our utmost, fail to save the pulp's life, (and fail we will in some cases,) we still have the resource at command of saving the tooth, minus its pulp, and also the right to glory in the fact that we have honestly striven to preserve what God intended never to be destroyed.

